# Does the Mind Wander When the Brain Takes a Break? Local Sleep in Wakefulness, Attentional Lapses and Mind-Wandering

**DOI:** 10.3389/fnins.2019.00949

**Published:** 2019-09-13

**Authors:** Thomas Andrillon, Jennifer Windt, Tim Silk, Sean P. A. Drummond, Mark A. Bellgrove, Naotsugu Tsuchiya

**Affiliations:** ^1^School of Psychological Sciences, Turner Institute for Brain and Mental Health, Monash University, Melbourne, VIC, Australia; ^2^School of Philosophical, Historical and International Studies, Monash University, Melbourne, VIC, Australia; ^3^School of Psychology, Deakin University, Melbourne, VIC, Australia; ^4^Murdoch Children’s Research Institute, Melbourne, VIC, Australia; ^5^Department of Paediatrics, University of Melbourne, Melbourne, VIC, Australia; ^6^Center for Information and Neural Networks (CiNet), National Institute of Information and Communications Technology (NICT), Osaka, Japan; ^7^Advanced Telecommunications Research Computational Neuroscience Laboratories, Kyoto, Japan

**Keywords:** sleep, physiology, performance, wakefulness, phenomenology

## Abstract

Sleep has been classically described as an all-or-nothing global phenomenon. However, recent research strongly suggests that this view requires tempering. Invasive and non-invasive recordings in animals and humans show that neural activity typically associated with sleep can locally occur during wakefulness. Although local sleep is defined neuronally, it has been associated with impaired performance during cognitive tasks. Comparatively, the phenomenology of local sleep (i.e., what it feels like when your brain is partially asleep) has been less explored. Taking into account the literature on the neuronal and behavioral profile of local sleep intrusions in wakefulness, we propose that occurrences of local sleep could represent the neural mechanism underlying many attentional lapses. In particular, we argue that a unique physiological event such as local sleep could account for a diversity of behavioral outcomes from sluggish to impulsive responses. We further propose that local sleep intrusions could impact individuals’ subjective experience. Specifically, we propose that the timing and anatomical sources of local sleep intrusions could be responsible for both the behavioral consequences and subjective content of attentional lapses and may underlie the difference between subjective experiences such as mind wandering and mind blanking. Our framework aims to build a parallel between spontaneous experiences in sleep and wakefulness by integrating evidence across neuronal, behavioral and experiential levels. We use the example of attention deficit hyperactivity disorder (ADHD) to illustrate how local sleep could explain complex cognitive profiles which include inattention, impulsivity, mind-wandering and mind-blanking.

## Introduction

Sleep and wakefulness have been traditionally considered as mutually exclusive states. At the behavioral level, sleep is characterized by a transient loss of responsiveness to the environment (Carskadon and Dement, 2005; Cirelli and Tononi, 2008). This behavioral unresponsiveness was found to correspond, at the physiological level, to specific patterns of brain activity. Notably, Non-Rapid Eye Movement (NREM) sleep, which amounts to 75–80% of the total time spent asleep in healthy adults (Ohayon et al., 2004; Carskadon and Dement, 2005), is characterized by the occurrence of high-amplitude slow oscillations. These so-called “slow waves” were initially described as alternations between moments of neuronal silencing and firing synchronized across the entire cortex (Steriade, 2003; Vyazovskiy and Harris, 2013).

Recent discoveries in animal and human sleep physiology have tempered the notion of slow waves as necessarily being a global physiological event (Nobili et al., 2012; Siclari and Tononi, 2017; Krueger et al., 2019). Within NREM sleep, some brain regions can show slow waves while others do not (Nir et al., 2011; Nobili et al., 2011). Such regional aspects of sleep activity had already been observed in certain animal species (e.g., dolphins who can enter unihemispheric sleep, sleeping in one brain hemisphere at a time) (Mascetti, 2016; Rattenborg et al., 2019) and in sleep pathologies (Terzaghi et al., 2009; Dodet et al., 2015; Castelnovo et al., 2016; Riedner et al., 2016). However, in the past few years, local sleep involving changes in sleep depth within NREM sleep has been robustly observed in individuals without sleep disorders (Huber et al., 2004; Nir et al., 2011). Even more striking is the observation of local sleep-like slow waves outside of NREM sleep, in wakefulness (Vyazovskiy et al., 2011; Hung et al., 2013; Bernardi et al., 2015; Quercia et al., 2018) or REM sleep (Funk et al., 2016; Bernardi et al., 2019). These slow waves are isolated (local in time) and spatially restricted (local in space) and consequently largely overlooked in standard classifications that focus on the global characteristics of sleep and wake states. The occurrence of local slow waves outside NREM sleep (i.e., in wakefulness or REM sleep) and the local modulation of the presence of slow waves within NREM sleep have been termed ‘local sleep’ (Box 1).

**Box 1.**

**Glossary:** Some of the terms used in this Perspective are widely used but not systematically defined and the same terms may be used by behavioral, phenomenological and/or neurophysiological approaches to refer to slightly different target phenomena. Below we define each term to the extent that is acceptable across different disciplines in order to facilitate further interdisciplinary work, both theoretical and empirical.

**Wakefulness:** Wakefulness is a state in which individuals can rapidly and reliably react to environmental demands. At the physiological level, wakefulness is characterized by a pattern of brain activity dominated by fast, low-amplitude, desynchronized oscillations.

**Sleep:** Sleep is a state behaviorally defined by a transient loss of responsiveness. To induce responses, stimuli have to be more intense than during wakefulness. The recovery of responsiveness is usually associated with a reversal to neural patterns of wake activity. Neuronal activity during sleep is dominated by slow, high-amplitude, synchronized oscillations in Non-Rapid Eye Movement (NREM) sleep and by low-amplitude, theta (4–7 Hz) and mixed-frequency oscillations in Rapid Eye Movement (REM) sleep.

**Local Sleep:** A concept introduced by Huber et al. (2004) (in sleep) and Vyazovskiy et al. (2011) (in wakefulness). In its most general sense, local sleep refers to transient, regional neurophysiological states showing a mixture of features characteristic of (i) wakefulness and sleep, (ii) different sleep stages (NREM and REM sleep), or (iii) different sleep depths (light or deep sleep). Local sleep is local both in time and space. As a relatively new concept in the sleep literature, its precise definition is likely to evolve with time.

**Microsleep:** Microsleep is classically defined as a global shift in neuronal activity from wakefulness to light NREM sleep for a duration of 5 to 14 s. Above 14 s, the individual is considered asleep. Below 5 s, the individual is considered awake but could potentially show signs of local sleep (< 5 s).

**Attentional lapse:** Attentional lapses refer to the redirection of an individual’s attention away from a specific task. These lapses are accompanied by a drop in objective performance as well as an increase in performance variability.

**Mind-wandering:** Mind-wandering is defined as spontaneous, dynamic and often associative thought. In the context of laboratory experiments, this is often operationalized as task- and/or stimulus independent thought. Mind-wandering can be considered as the phenomenological dimension of a large group of attentional lapses.

**Mind-blanking:** Mind-blanking refers to subjective reports of reduced awareness and a temporary absence of thought (empty mind) or lack of memory for immediately past thoughts. Mind-blanking can be considered as the phenomenological dimension of a distinct kind of attentional lapse compared to mind-wandering.

**ADHD:** Attention deficit hyperactivity disorder (ADHD) is a common neurodevelopmental disorder with a worldwide prevalence of approximately 5%. The diagnosis is based on age-inappropriate levels of inattention, hyperactivity and/or impulsivity.

### Behavioral and Neuronal Effects of Local Sleep

Local sleep during wakefulness was originally defined based on the identification of a hallmark of NREM sleep within wakefulness: sleep slow waves (Vyazovskiy et al., 2011). These sleep slow waves can be observed in invasive intracranial recordings [local field potentials (LFP), electrocorticogram (ECoG)] or non-invasive scalp electroencephalography (EEG) in the form of high-amplitude, slow oscillations (< 4 Hz). At the level of individual neurons, these slow oscillations involve a brief episode of neuronal silencing (OFF period) followed by synchronous activation of neighboring neurons (Steriade, 2003; Vyazovskiy and Harris, 2013). OFF periods appear as the most unequivocal definition of local sleep intrusions during wakefulness as they directly show an increase in local synchrony and an interruption of neural activity similar to that which can be observed during sleep. However, these OFF periods cannot be recorded non-invasively.

Investigations in humans have therefore focused on the detection of local high-amplitude slow oscillations, which typically accompany OFF periods (Steriade, 2005). However, the exact frequency bands used to detect these waves differ from one study to another, with some studies focusing on the theta range (Hung et al., 2013; Bernardi et al., 2015), others on the delta range (Quercia et al., 2018), and yet others on a combination of the theta and delta range (Vyazovskiy et al., 2011; Nir et al., 2017). It is unclear if these so-called delta and theta waves refer to distinct events. Indeed, during normal sleep, slow waves have complex frequency profiles (e.g., a mixture of a delta component with faster oscillations) (Siclari et al., 2014; Halász, 2016). Further, there can be considerable variability when estimating the frequency of short-lived, isolated slow waves. Adding to the difficulty of comparing existing methods is the fact that the basic physiological properties of local sleep events in wakefulness are yet to be determined (topographical distribution, spatial extent, peak frequency, duration, etc). These parameters have been studied in sleep (Riedner et al., 2007; Siclari et al., 2014) but not in wakefulness. Existing studies suggest that local slow waves occur in both frontal and parietal regions (Vyazovskiy et al., 2011; Hung et al., 2013; Quercia et al., 2018), with a larger number of waves in parietal regions (Vyazovskiy et al., 2011; Hung et al., 2013).

**FIGURE 1:**
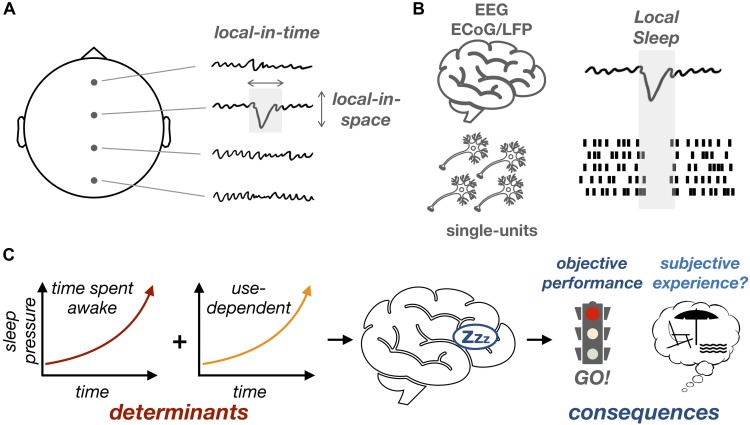
Can local sleep in wakefulness affect behavior and subjective experience? **(A)** Isolated sleep-like slow waves (which are local in time and space) can be observed during wakefulness. **(B)** These slow waves can be observed in scalp EEG or intracranial recordings (ECoG/LFP, top) and are associated at the level of single-unit recordings (bottom) with episodes of neuronal silencing. **(C)** Local sleep increases with time spent awake, even more so in brain regions involved in a given recurrent task (use-dependent). The timing and location of local sleep occurrences could determine their impact on objective performance and, possibly, subjective experience.

In summary, local sleep can be defined, at the level of neuronal networks, by the brief local appearance of events similar to sleep slow waves (delta and/or theta waves) which are in turn associated, at the level of single-neuron activity, with episodes of neuronal silencing (Figure 1). Local sleep is thus different from the concept of microsleep (Box 1) as it is defined by the local occurrence of a specific pattern of neural activity (slow wave) for, potentially, a much shorter period of time than microsleep. As is the case for microsleep, however, local sleep can be reliably induced by sleep deprivation. First, when examining scalp EEG, the power in the delta/theta band (corresponding to sleep slow waves) increases with time spent awake (Cajochen et al., 1995), a phenomenon thought to reflect the build-up of sleep pressure (Finelli et al., 2000) and potentially mediated by functional and structural changes in neuronal networks (Tononi and Cirelli, 2014). More recently, it has been shown that this increase in the power of slow oscillations is accompanied by the occurrence of isolated waves that bear a striking resemblance to sleep oscillations (Vyazovskiy et al., 2011; Hung et al., 2013; Bernardi et al., 2015; Quercia et al., 2018).

Interestingly, the occurrence of local sleep intrusions varies as a function of both the time spent awake and the degree to which a particular brain region has been activated by environmental demands (e.g., a specific task) (Hung et al., 2013; Bernardi et al., 2019). A recent study linked the occurrence of local slow waves during sleep with neuronal plasticity and learning (Quercia et al., 2018). Using a spatial memory task, Quercia et al. (2018) showed that local sleep occurrences in wakefulness are predictive of errors but can also be explained by learning-related processes. This is particularly interesting since similar use-dependent or learning-dependent regional modulations of slow waves have been observed during sleep (Huber et al., 2004; Bernardi et al., 2015). Altogether, these results support a link between local sleep and neuronal plasticity in both wakefulness and sleep. In a nutshell, the recruitment of a neuronal network by a given task would fatigue this network, particularly when the network is subject to plastic changes. This neuronal fatigue would translate in local sleep occurrences, which would negatively impact behavior in a region-specific, task-specific fashion.

At the same time, it is unlikely that extensive usage of local neural circuitry is the sole predictor of local sleep occurrences. Rather, it is more likely that local sleep is modulated by a combination of use-dependent and circadian factors. Sleep in general is known to be influenced by both time spent awake and circadian processes (Borbély, 1982). Consistent with this view, previous research has demonstrated the existence of local modulations of brain activity during the day, in response to both circadian rhythms and sleep pressure (Muto et al., 2016). However, this circadian viewpoint has not been taken into account by the majority of studies on local sleep and represents an important future research direction.

Although local sleep has been primarily defined at the physiological level, its occurrence during wake has been associated with impaired task performance: trials preceded by local slow waves typically show slower reaction times and increased error rates (Vyazovskiy et al., 2011; Hung et al., 2013; Bernardi et al., 2015; Nir et al., 2017; Quercia et al., 2018). These performance decrements were proposed to result from the transient period of neuronal silencing associated with local slow waves (Vyazovskiy et al., 2011). Indeed, during sleep, episodes of neuronal silencing accompanying slow waves have been associated with perturbations of sensory encoding and information processing (McCormick and Bal, 1994) and it has been proposed that sleep slow waves could be the mechanism explaining sensory isolation (Schabus et al., 2012; Andrillon et al., 2016) and loss of consciousness (Tononi and Massimini, 2008) in NREM sleep. Importantly, the behavioral consequences of local sleep in rodents engaged in a specific task are region-specific: local sleep intrusions occurring in the neural networks involved in the task were more likely to lead to behavioral impairments (Vyazovskiy et al., 2011). Likewise, in humans, local sleep occurrences led to task-specific behavioral impairments (Hung et al., 2013; Bernardi et al., 2015).

### Impact of Local Sleep on Phenomenology

Local modulations of sleep-like activity not only impact behavioral performance during wakefulness, but might also affect the contents or structure of subjective experience during sleep. Recent research, relying on subjective reports and serial-awakening paradigms (Siclari et al., 2013), has emphasized the diversity of sleep-related subjective experiences, ranging from immersive, narratively complex and often emotionally intense dreams to isolated thoughts and imagery, to a form of minimal subjective experience that lacks specific thought contents and imagery (Windt, 2015; Windt et al., 2016). We propose that local aspects of sleep could explain the emergence of different sleep-related experiences. For example, local modulations of slow-wave activity within NREM and REM sleep have been associated with the occurrence of dreams (Siclari et al., 2016). Specifically, a decrease in the number of slow waves (which can be interpreted as a regional reduction of sleep depth) in parietal regions is associated with subjective reports of dreaming upon awakening, although the causal nature of this relationship is debated (Boly et al., 2017; Wong et al., 2019). The spatio-temporal properties of the decrease in slow-wave activity are predictive not only of the occurrence of dreams but also of their content (Siclari et al., 2016) (e.g., a decrease in slow-wave activity in motor regions was associated with more reports of movements during dreams). These results suggest that local slow waves could act as a functional switch enabling or disabling specific cognitive processes during sleep, with direct consequences on oneiric contents (Siclari et al., 2016) but also the ability to process external stimuli (Andrillon et al., 2016, 2017; Tamaki et al., 2016; Blume et al., 2018).

Local sleep in wakefulness could be associated with similar changes in subjective experience. Indeed, spontaneous experiences in wakefulness and sleep share similar properties at the phenomenological level. Wakeful cognition is a highly dynamic process and fluctuations in spontaneous experience forming the so-called “stream of thought” (James, 1890) are at the core of mind-wandering research (Smallwood and Schooler, 2015) (Box 1). The sampling of conscious experiences during the day (Wamsley, 2013; Smallwood and Schooler, 2015), an approach similar to that used in dream research (Horikawa et al., 2013; Siclari et al., 2013), reveals that individuals spend on average 30–50% of their time thinking about something other than the task at hand (Killingsworth and Gilbert, 2010; Seli et al., 2013).

It has been suggested that dreaming and mind wandering could be placed on a continuum (Fox et al., 2013; Domhoff, 2018). First, dreaming and mind wandering seem supported by overlapping neural networks, including the Default Mode Network (DMN) (Fox et al., 2013). Second, from a phenomenological perspective, dreams are at core immersive, involving a simulated world centered on a simulated self (Windt, 2015; Windt et al., 2016). Similarly, daydreams tend to be focused on self-related concerns (D’Argembeau, 2018) and often contain vivid audio-visual imagery as well as emotions (Foulkes and Fleisher, 1975). Yet, daydreams lack the “here and now” quality of sleep dreams: in wakefulness, we don’t lose touch with our actual environments or feel present in imagined ones as completely as we do in sleep-dreams. In this sense, dreams seem to be an intensified form of mind wandering.

Other sleep-experiences lack the immersive character of dreaming and hence have been termed dreamless sleep experiences. A subgroup of these is characterized by an absence of reportable content; these seem to involve simple or perhaps even minimal forms of subjective experience in which specific forms of thought contents or imagery are lacking (Windt, 2015; Windt et al., 2016). This subgroup of dreamless sleep experiences appears close to what has been termed mind-blanking in wakefulness, that is to say episodes in which individuals report a lack of conscious awareness (Ward and Wegner, 2013).

If sleep- and wake-related spontaneous experiences share similar properties at the phenomenological level, are they also related at the physiological level? More precisely, can local sleep explain the presence or absence (as well as content) of spontaneous experiences not just in sleep, but also in waking? This tantalizing question will need further investigation but initial results are promising. First of all, while the local decrease in slow-wave activity over parietal regions correlates with the occurrence of spontaneous experiences during sleep (dreams), a local increase during wakefulness over the same regions correlates with a reduction in spontaneous thoughts (Perogamvros et al., 2017). These results are in line with the involvement of the parietal cortex in multimodal integration (Alais et al., 2010) and consciousness (Koch et al., 2016; Boly et al., 2017). Other physiological indexes of vigilance have shown a link between fluctuations in attention or subjective experience and sleepiness. Attentional lapses in general have been associated with a diminution of noradrenergic activity using fMRI or pupillometry (Mittner et al., 2014, 2016), a proxy for noradrenergic activity (Varazzani et al., 2015; Joshi et al., 2016). This is particularly interesting as Noradrenaline (NA) is one of the key neuromodulators allowing sleep/wake transitions (Siegel, 2004) but also plays a central role in the modulation and orientation of attention (Sara, 2009; Sara and Bouret, 2012; Thiele and Bellgrove, 2018). At the phenomenological level, mind-blanking in particular has been associated with a reduction in pupil diameter and increase in subjective sleepiness (Unsworth and Robison, 2018). Here, we go one step further by hypothesizing that fluctuations in spontaneous experiences during wake not only correlate with states of low vigilance but actually share common neuronal mechanisms with sleep-related spontaneous experiences.

### Local Sleep as a Comprehensive Model of Attentional Lapses

**FIGURE 2:**
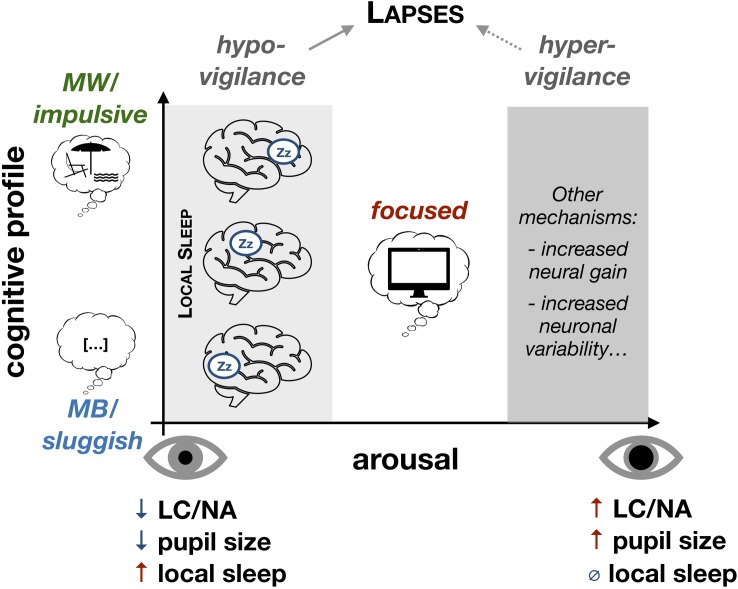
Local sleep as a comprehensive model of attentional lapses under low levels of arousal. Under low levels of arousal (hypo-vigilance), the likelihood that local sleep intrusions occur increases. The spatial properties of these local sleep intrusions (which brain regions are affected) would determine the cognitive profile of attentional lapses (impact on objective performance and subjective experience). Global fluctuations of arousal could be determined by the level of activation of the Locus Coeruleus (LC) and the concentration in Noradrenaline (NA). Spatio-temporal properties of local sleep would further describe the cognitive profile of attentional lapses. Importantly, our model does not exclude the possibility that, under high levels of arousal (hyper-vigilance), objective performance and subjective performance can be affected by other neurophysiological mechanisms. MW, mind-wandering; MB, mind-blanking.

We propose a multi-level model of attentional lapses from neurophysiology to behavior and phenomenology. In our framework, local sleep intrusions represent a unifying physiological mechanism that could not only predict the occurrence of attentional lapses but also describe these lapses in terms of their behavioral consequences and phenomenological properties. Other markers of low arousal have been proposed to predict attentional lapses. In particular, pupil size can predict the occurrence of attentional lapses and mind-wandering (Mittner et al., 2014; van den Brink et al., 2016). However, pupil size is a global, systemic index of arousal [although pupil diameter can be linked with specific components of behavior (van Kempen et al., 2019)] and our local sleep framework could help move beyond global indexes to offer a regional marker predictive of specific behavioral impairments (Figure 2). Interestingly, in this framework, a single physiological event can explain phenomena that have often been presented as opposed: impulsivity vs. sluggishness and mind-wandering vs. mind-blanking.

Previous studies investigating local sleep intrusions during wake focused mostly on task and region-specific effects, after forcing participants to continuously perform a single task [e.g., (Hung et al., 2013; Bernardi et al., 2015)]. The goal of such paradigms is to fatigue circumscribed neural networks engaged in the task, increasing the likelihood of local sleep intrusions which would then result in errors on that particular task. In this type of paradigm, local sleep occurring in a given set of brain regions is expected to have the same behavioral outcomes. However, most everyday tasks require complex behavior and engage different brain regions. Participants performing these tasks are prone to multiple types of action failures (Smith and Ratcliff, 2004), whose relationship with sleepiness are often unclear (Anderson and Horne, 2006; Anderson et al., 2010; Drummond et al., 2012). An alternative method is to observe the occurrence of local sleep, attentional lapses and sleep-like activity during wakefulness following sleep deprivation (Hung et al., 2013; Bernardi et al., 2015) or even under normal conditions (Quercia et al., 2018). Here, we propose that where and when local sleep occurs could predict not only the timing of errors but the type of error being made in a specific task.

In particular, local sleep occurrences could explain the occurrence of seemingly opposed behavioral failures such as omission errors (failure to produce a response) and commission errors (failure to control, select or suppress a response). The standard view is that commission errors are associated with executive control failure, whereas omission errors would rather mark the failure to maintain a more basic form of vigilance (Ballard, 2001; O’Connell et al., 2008; Van Schie et al., 2012). This view would predict that omission and commission errors occur in different contexts of vigilance. Research on the effects of sleep deprivation showed, however, that sleep loss could actually result in both impulsivity and sluggishness (Drummond et al., 2006; Anderson and Platten, 2011). According to our framework, the same neural mechanism (local sleep) occurring in different parts of the brain could explain both commission and omission errors associated with sleepiness. This is supported by previous research in humans showing that local sleep-like activity in perceptual areas leads to slower reaction times in a visual psychomotor vigilance task (Nir et al., 2017), whereas slow waves in motor areas lead to decreased motor performance and slow waves in frontal areas lead to inhibition errors (Bernardi et al., 2015).

Next, we hypothesize that local sleep can account not only for decreases in objective behavioral performance but also for changes in individuals’ subjective experience. The impact of local sleep on subjective experience has been less frequently studied (Perogamvros et al., 2017) than its consequences on objective performance (Hung et al., 2013; Bernardi et al., 2015; Nir et al., 2017; Quercia et al., 2018). Previous work does acknowledge, however, that sleep restriction can lead to increased distractibility in various ways (e.g., increased sensitivity to environmental vs. endogenous distractors) (Anderson et al., 2010). A more systematic approach to record and analyze subjective experience is needed. Based on current findings, we suggest that it will be useful to distinguish: (i) lapses induced by environmental stimuli (or external distractions); (ii) spontaneous thoughts (mind-wandering); (iii) task-related interferences (e.g., performance monitoring), and (iv) mind-blanking. We stress here the importance of including the dimension of mind-blanking. By contrast, mind-wandering research usually assumes that the mind wanders somewhere rather than nowhere (Ward and Wegner, 2013; Smallwood and Schooler, 2015), leading to a comparative neglect of mind blanking as compared to mind wandering.

Importantly, it has been previously suggested that these different types of subjective experiences correspond to different neural mechanisms and different levels of arousal. Mind-wandering has been proposed to be associated with a high level of arousal and fleeting or racing thoughts, whereas mind-blanking has been associated with decreased vigilance and low arousal (Mittner et al., 2016; Unsworth and Robison, 2018). However, this view is at odds with the fact that mind-wandering can occur in states or individuals with high sleep pressure (Braboszcz and Delorme, 2011; Carciofo et al., 2014; Poh et al., 2016) and typically increases when environmental demands decrease (e.g., easy or unmotivating tasks) (Antrobus et al., 1966). We offer here an alternative explanation to reconcile these views (Figure 2). We propose that the occurrence of mind-wandering is fully compatible with a low level of arousal and a state of subjective and objective fatigue and that local sleep intrusions could mechanistically account for both mind-wandering and mind-blanking. Similarly, as for behavioral outcomes, the subjective experience associated with a given local sleep occurrence would depend on the anatomical location of these local sleep intrusions, which is a well-defined, testable hypothesis.

### ADHD: A Test Case for Our Novel Framework

Local sleep could also represent a powerful explanatory account of inter-individual differences in attentional lapses in conditions such as attention deficit hyperactivity disorder (ADHD). ADHD is a common neurodevelopmental disorder that affects 1–3% of adults and 3–7% of children (Loe and Feldman, 2007; Ebejer et al., 2012). It is characterized by age-inappropriate levels of inattention, hyperactivity and/or impulsivity, which result in substantial challenges in life (decreased socioeconomic status, increased lifetime comorbid psychiatric disorders and higher rates of premature death) (Dalsgaard et al., 2015). ADHD has a high genetic heritability (74%) (Faraone and Larsson, 2019) and is associated with changes in brain anatomy and neural communication (Faraone et al., 2000). However, the neural mechanisms underpinning the inattention and impulsivity which characterize the disorder remain unclear.

Key behavioral attributes of ADHD, established across numerous tasks, are the increase in omission errors, greater intra-individual variability in response timing, as well as more infrequent extremely slow responses (Bellgrove et al., 2005; Johnson et al., 2007a, b). At the phenomenological level, a recent study showed that both teenagers and adults diagnosed with ADHD also differ from non-ADHD individuals, showing increased levels of mind-blanking (Van den Driessche et al., 2017), at the expense of both attentive states and mind-wandering.

This behavioral and experiential fingerprint of ADHD involving sluggishness and mind-blanking portrays ADHD as hypo-vigilant. This could seem at odds with the popular association between ADHD and hyperactivity. However, the association between ADHD and hypo-vigilance fits well with the frequent observation of sleep disturbances in ADHD adults and children (Konofal et al., 2010; Hvolby, 2015; Virring et al., 2016; Gregory et al., 2017; Hiscock and Sciberras, 2019; Sciberras et al., 2019; Silk, 2019). In fact, as many as 73% of children with ADHD experience mild to severe sleep issues (Sung et al., 2008). Children diagnosed with ADHD who exhibit sleep problems also tend to have more severe ADHD symptoms as well as poorer daily functioning compared to ADHD children without sleep problems. In addition, treatments used in ADHD (e.g., methylphenidate) are also used to curb excessive daytime sleepiness in narcolepsy (Hvolby, 2015). Methylphenidate acts on the noradrenergic pathway, which controls attention and sleep/wake regulation (Siegel, 2004; Sara and Bouret, 2012), once again suggesting a mechanistic link between the regulation of attention and modulations of sleep/wake activity.

A key question in the ADHD literature is therefore whether these sleep problems are mere correlates of the attentional disorder or whether they could be at the root of the behavioral features of ADHD. In favor of the latter, it has been hypothesized that ADHD symptoms arise from a dysregulation of arousal (Geissler et al., 2014). Accordingly, ADHD patients may have elevated arousal levels at night, which could delay sleep onset and perturb sleep. As a consequence, these individuals would feel sleepy during the day, which could translate into an increase in local sleep intrusions and give rise to a pattern of sluggishness and impulsivity, mind-wandering and mind-blanking.

Thus, given that individuals with ADHD exhibit, when compared to healthy controls: (1) greater attentional lapses that affect task performance; (2) higher rates of mind-blanking; and (3) more sleep disturbances than healthy controls, local sleep could provide an interesting and largely overlooked neural mechanism for the behavioral phenotype in ADHD. This novel framework in ADHD research could also improve current therapeutic approaches, as current medications can improve daytime performance but can also perturb sleep (Hvolby, 2015). A more systematic investigation of the impact of ADHD treatments on nighttime sleep and daytime sleepiness could help reassess the costs and benefits of these treatments. Finally, current ADHD therapeutic approaches could be complemented with a focus on the improvement of both sleep quantity and quality.

## Discussion

We outline here testable hypotheses regarding the relationship between the occurrence of local sleep and the behavioral and phenomenological profiles of attentional lapses. We hypothesize that local sleep occurrences could not only predict attentional lapses but also the type of lapse depending on the anatomical location of local sleep intrusions. Our model could lead to a better understanding of phenomena such as mind wandering and mind blanking. Indeed, mind wandering has been associated with the recruitment of the DMN (Mason et al., 2007). Interestingly, it has been argued that similar patterns of brain activation can be accounted for by drowsiness in resting-state functional Magnetic Resonance Imaging (fMRI) (Tagliazucchi and Laufs, 2014). In addition, it seems that DMN’s activation is mirrored by a deactivation of attentional networks such as the dorsal attention network (Mittner et al., 2016). Local sleep occurrences within attentional networks could be the physiological trigger leading to the deactivation of attentional networks and the recruitment of the DMN, the combination of both resulting in an episode of mind wandering. Conversely, occurrences of local sleep within the DMN could prevent the emergence of spontaneous thoughts (mind wandering), leading to a state of mind blanking. Accordingly, previous findings (Perogamvros et al., 2017) show that an increase in slow-wave power in regions overlapping with the DMN can reduce endogenous thoughts in both wakefulness and sleep. Finally, as during sleep, the set of brain regions affected by an episode of local sleep could be predictive of the specific content of the associated thoughts. For example, local sleep occurrences in prefrontal cortices could be associated with a lack of awareness and agency such as in dreaming (Nir and Tononi, 2010; Voss et al., 2014). Local sleep occurrences in perceptual or associative areas, such as the “hot zone” identified by Siclari et al. (2016) (including sensory areas, precuneus, posterior cingulate), could lead to the occurrence of minimal subjective experience devoid of specific forms of imagery and thought contents (i.e., mind blanking) similar to white dreams during sleep (Windt et al., 2016; Fazekas et al., 2019).

We further propose that local sleep could account for inter-individual differences in the frequency and type of attentional lapses. In particular, focusing on individuals with ADHD, we hypothesize that the reported increase in mind-blanking, at the expense of both mind-wandering and a focused state (Van den Driessche et al., 2017), may be traced back to an increase in local sleep intrusions during the day. This larger propensity to enter mixed sleep/wake states could in turn be related to the sleep disturbances that often accompany the disorder. By showing that individuals with ADHD show more instances of local sleep, we could offer a more exhaustive account of their symptoms, which links daytime performance with disruptions of the restorative function of sleep.

Importantly, we do not argue here that all forms of attentional lapses can be explained by sleepiness and local sleep intrusions, nor that changes in subjective experience such as mind-wandering and mind-blanking are always associated with local sleep. It is of course possible that attentional lapses with similar behavioral consequences to those discussed here can occur in contexts of hyper-arousal rather than hypo-vigilance (Figure 2). Nonetheless, we propose that attentional lapses associated with hypo-vigilance might be more frequent.

Although promising, this framework needs to be further established. It has already been suggested that local sleep intrusions, depending on their location, can affect specific aspects of wake behavior (Vyazovskiy et al., 2011; Hung et al., 2013; Bernardi et al., 2015; Nir et al., 2017; Quercia et al., 2018). However, previous findings were obtained in different tasks or studies, or investigated the correlation between local sleep and performance across trials and individuals rather than on the single-trial level. Similarly, the link between local sleep and subjective experience is suggested by previous research but these studies focused mostly on subjective experiences occurring during sleep. Although there is a phenomenological similarity between mind-wandering, mind-blanking and sleep-related experiences and while it is suggestive that both mind-wandering and mind-blanking occur under conditions of low arousal (Braboszcz and Delorme, 2011; Poh et al., 2016; Unsworth and Robison, 2018), this needs to be established through simultaneous phenomenological and neural recordings. Furthermore, it is yet to be determined whether spatial properties of local sleep intrusions could predict changes in spontaneous experience in a region-specific fashion.

Finally, the conditions under which local sleep can be observed are yet unclear. Initial studies have shown local sleep intrusions following extensive sleep deprivation, with the first intrusions occurring only after a few hours of sleep restriction (Hung et al., 2013; Bernardi et al., 2015; Nir et al., 2017). A recent study, however, showed that local sleep can also occur during an experimental task after a normal night of sleep (Quercia et al., 2018). Is local sleep then an abnormal event, occurring when the brain is pushed to its limits or does it represent a normal component of the healthy brain’s physiology? Does local sleep affect each of us or is it constrained to very specific contexts or sub-populations? To answer these questions, it is necessary to perform interdisciplinary studies, combining the methods and approaches of sleep, vigilance and attention research. For example, paradigms established in attention research (Robertson et al., 1997; O’Connell et al., 2009; Dockree et al., 2017) and seeking to explore the sub-processes of sensory processing and decision making can be modified to allow the probing of subjective experience with a comprehensive taxonomy of spontaneous cognition (Smallwood and Schooler, 2015). The parallel recording of brain activity and in particular of EEG and pupillometry could then allow to map changes at the behavioral and experiential levels to physiological markers of local sleep and arousal (Bernardi et al., 2015; van Kempen et al., 2019). Exploring local sleep with complementary brain imaging techniques such as Magnetoencephalography (MEG) or fMRI could help understand how local a given local sleep event is and how the occurrence of local sleep in one region can functionally affect connected regions. Our framework is designed to support a versatile methodology that could explore the impact of local sleep in various contexts (with or without sleep deprivation, with or without pharmacological treatments, in response to circadian manipulation, etc) and different populations (healthy individuals, ADHD, insomnia, etc).

## Author Contributions

All authors contributed to the review and discussion of the literature and to the proposition of future directions.

## Conflict of Interest Statement

The authors declare that the research was conducted in the absence of any commercial or financial relationships that could be construed as a potential conflict of interest.
